# The mitochondrial genome of *Vulgichneumon* sp. (Hymenoptera: Ichneumonidae)

**DOI:** 10.1080/23802359.2019.1660922

**Published:** 2019-09-06

**Authors:** Yimeng Chen, Yiying Chen, Fengqin Cao, Zhifu Cui

**Affiliations:** a College of Plant Protection, Hainan University, Haikou, China;; b Hainan State Farms Academy of Sciences Group Co. Ltd., Haikou, China

**Keywords:** Mitochondrial genome, Ichneumoninae, phylogenetics

## Abstract

The *Vulgichneumon* sp. belongs to the subfamily Ichneumoninae of Ichneumonidae. The mitogenome (GenBank accession number: MN178162) of *Vulgichneumon* sp. was sequenced, the first representative of the mitogenome of the subfamily. The nearly complete mitogenome is 15,306 bp totally, consisting of 13 protein-coding genes, 2 rRNAs and 22 transfer RNAs. The nucleotide composition biases toward A and T, which together made up 83.6% of the entirety. Bayesian inference analysis supported the monophyly of Evanioidea, Chalcidoidea, Platygastroidea, Proctotrupoidea, Ceraphronoidea, and Ichneumonoidea. This result also suggested that the clade that contains other six superfamilies was the sister group to the clade that consist of Trigonaloidea, Megalyroidea and Ichneumonoidea.

## Introduction

The Hymenoptera (ants, bees, and wasps) comprise one of the largest (LaSalle and Gauld 1993) and most biologically diverse group of insects (Gaston 1991) containing both eusocial and parasitic groups.

The specimens of *Vulgichneumon* sp. used for this study were collected from Weichang Manchu and Mongol Autonomous County of Heibei Province by Yimeng Chen and identified by Yimeng Chen. Specimens are deposited in the College of Plant Protection, Hainan University with the deposit number: HN NO.V20180926. The total genomic DNA was extracted from the whole body (except head) of the specimen using the QIAamp DNA Blood Mini Kit (Qiagen, Germany) and stored at −20 °C until needed. The mitogenome was sequenced in Allwegene Technology Limited Company with the sequenced number: AWGT19042503-CC. The nearly complete mitogenome of *Vulgichneumon* sp. is 15,306 bp (GenBank accession number: MN178162). It encoded 13 PCGs, 22 tRNA genes, 2 rRNA genes and were similar with related reports before (Cha et al. 2007; Wei et al. 2009; Tan et al. 2011; Eimanifar et al. 2017). The nucleotide composition of the mitogenome was biased toward A and T, with 83.6% of A + T content (A = 41.2%, T = 42.4%, C = 10.4%, G = 6.0%). The A + T content of PCGs, tRNAs, and rRNAs is 82.0, 85.6, and 86.5%, respectively. The total length of all 13 PCGs of *Vulgichneumon* sp. is 10,966 bp. Six PCGs (*NAD2, COI, ATP8, NAD3, NAD4,* and *NAD6*) initiated with ATT codons and four PCGs (*COIII*, *ATP6*, *NAD5,* and *CYTB*) initiated with ATG codons, *NAD1, NAD4L,* and *COII* initiated with ATA as a start codon. Twelve PCGs used the typical termination codons TAA except *NAD4* used T in *Vulgichneumon* sp.

**Figure 1.**
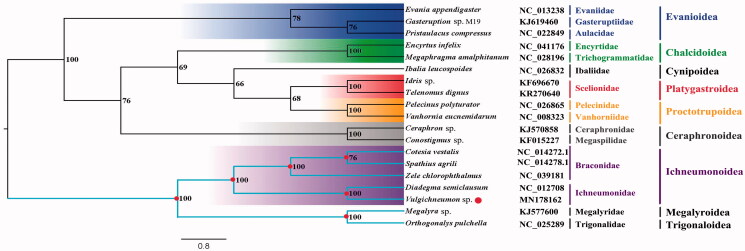
Bayesian phylogenetic tree of 19 Hymenoptera species. The posterior probabilities are labeled at each node. Genbank accession numbers of all sequence used in the phylogenetic tree have been included in Figure 1 and corresponding to the species names.

Phylogenetic analysis was performed based on the nucleotide sequences of 13 PCGs from 19 Hymenoptera species. Bayesian (BI) analysis generated the phylogenetic tree topologies based on the PCGs matrices (Figure 1). According to the phylogenetic result, the monophyly of Evanioidea, Chalcidoidea, Platygastroidea, Proctotrupoidea, Ceraphronoidea, and Ichneumonoidea were supported. The Megalyroidea was sister to Trigonaloidea and then Ichneumonoidea was assigned to the sister group to the clade of Megalyroidea + Trigonaloidea and the clade that contains other six superfamilies was the sister group to the clade that consists of Trigonaloidea, Megalyroidea, and Ichneumonoidea. This result that the phylogenetic relationship among Trigonaloidea, Megalyroidea, and Ichneumonoidea are consistent with the phylogenetic result of the previous study (Mao et al. 2015). The nearly complete mitogenome of *Vulgichneumon* sp. could provide important information for the further studies of Hymenoptera phylogeny.

## References

[CIT0001] Cha SY, Yoon HJ, Lee EM, Yoon MH, Hwang JS, Jin BR, Han YS, Kim L 2007 The complete nucleotide sequence and gene organization of the mitochondrial genome of the bumblebee, *Bombus ignitus* (Hymenoptera: Apidae). Gene. 392:206–220. 17321076 10.1016/j.gene.2006.12.031

[CIT0002] Eimanifar A, Kimball RT, Braun EL, Fuchs S, Grünewald B, Ellis JD 2017 The complete mitochondrial genome of *Apis mellifera meda* (Insecta: Hymenoptera: Apidae). Mitochondr DNA B. 2:268–269. 10.1080/23802359.2017.1325342 PMC7800517 33473795

[CIT0003] Gaston KJ 1991 The magnitude of global insect species richness. Conserv Biol. 5:283–296.

[CIT0004] LaSalle J, Gauld ID 1993 Hymenoptera and biodiversity. Wallingford, UK: CAB International.

[CIT0005] Mao M, Gibson T, Dowton M 2015 Higher-level phylogeny of the Hymenoptera inferred from mitochondrial genomes. Mol Phylogenet Evol. 84:34–43. 25542648 10.1016/j.ympev.2014.12.009

[CIT0006] Tan H, Liu G, Dong X, Lin R, Song H, Huang S, Yuan Z, Zhao G, Zhu X 2011 The complete mitochondrial genome of the Asiatic cavity-nesting honeybee *Apis cerana* (Hymenoptera: Apidae). PLoS One. 6:e23008. 21857981 10.1371/journal.pone.0023008 PMC3155526

[CIT0007] Wei S, Shi M, He J, Sharkey M, Chen X 2009 The complete mitochondrial genome of *Diadegma semiclausum* (Hymenoptera: Ichneumonidae) indicates extensive independent evolutionary events. Genome. 52:308–319. 19370087 10.1139/g09-008

